# High-throughput cultivation and screening platform for unicellular phototrophs

**DOI:** 10.1186/s12866-014-0239-x

**Published:** 2014-09-16

**Authors:** Ulrich M Tillich, Nick Wolter, Katja Schulze, Dan Kramer, Oliver Brödel, Marcus Frohme

**Affiliations:** Molecular Biotechnology and Functional Genomics, Technical University of Applied Sciences Wildau, Hochschulring 1, 16-2001, D-15745 Wildau, Germany; Institute of Biology, Humboldt-University Berlin, Berlin, Germany; Cyano biotech GmbH, Magnusstrasse 11, D-12489 Berlin, Germany

**Keywords:** Cyanobacteria, *Synechocystis*, HTS, High throughput, Automated cultivation, Liquid handling

## Abstract

**Background:**

High-throughput cultivation and screening methods allow a parallel, miniaturized and cost efficient processing of many samples. These methods however, have not been generally established for phototrophic organisms such as microalgae or cyanobacteria.

**Results:**

In this work we describe and test high-throughput methods with the model organism *Synechocystis sp.* PCC6803. The required technical automation for these processes was achieved with a Tecan Freedom Evo 200 pipetting robot. The cultivation was performed in 2.2 ml deepwell microtiter plates within a cultivation chamber outfitted with programmable shaking conditions, variable illumination, variable temperature, and an adjustable CO_2_ atmosphere. Each microtiter-well within the chamber functions as a separate cultivation vessel with reproducible conditions. The automated measurement of various parameters such as growth, full absorption spectrum, chlorophyll concentration, MALDI-TOF-MS, as well as a novel vitality measurement protocol, have already been established and can be monitored during cultivation. Measurement of growth parameters can be used as inputs for the system to allow for periodic automatic dilutions and therefore a semi-continuous cultivation of hundreds of cultures in parallel. The system also allows the automatic generation of mid and long term backups of cultures to repeat experiments or to retrieve strains of interest.

**Conclusions:**

The presented platform allows for high-throughput cultivation and screening of *Synechocystis sp.* PCC6803. The platform should be usable for many phototrophic microorganisms as is, and be adaptable for even more. A variety of analyses are already established and the platform is easily expandable both in quality, i.e. with further parameters to screen for additional targets and in quantity, i.e. size or number of processed samples.

**Electronic supplementary material:**

The online version of this article (doi:10.1186/s12866-014-0239-x) contains supplementary material, which is available to authorized users.

## Background

High-throughput Screening (HTS) is a growing field which began in the mid 90’s along with the wide scale adoption of 96-well microtiter plates (MTPs). Its impact has been especially huge in pharmaceutical research, where libraries of numerous chemicals are screened for potential new drug candidates (compound screening) [1,2]. Modern HTS systems are often incorporated into liquid handling systems (pipetting robots), which allow automation, and massive increase in throughput of steps traditionally performed by trained lab technicians [3,2,4]. For many fields of pharmaceutical and biotechnology research the strong advances in recent decades would not have been possible without massively parallel processing of samples allowed by HTS. The throughput, and thus the amount of wells in the MTPs has been steadily increasing, while the volumes of the assays have been steadily dropping. Modern ultra HTS (uHTS) works with up to 3456 wells per plate, assay volumes at the picoliter level, and throughputs that can easily exceed 100,000 assays per day [5].

HTS is not well established for photothropic microorganisms however, though some systems for screening of microalgae based on FACS devices have been demonstrated [6,7]. These systems select cells with the desired characteristics out of a starting pool, and then validate the results of selected strains/organisms after upscaling. While this approach is straightforward and efficient, it is not as flexible as “traditional” HTS systems based on a liquid handling platform. It can only be used if a selection with a FACS is possible, which greatly limits the amount of parameters that can be screened. For many desirable traits (e.g. high expression/activity of a certain protein) FACS based methods are often not applicable. Furthermore, it is limited to the screening of single cells under the artificial conditions of the FACS but those single cells often behave different in real cultures. This makes it mandatory to screen cultures under conditions that better mimic later production processes. Besides, some screening targets might also require the strains to be continuously monitored during their growth, and various parameters measured in parallel (e.g. good growth with continuous secretion of a target compound). Therefore high throughput cultivation systems coupled with flexible analytic systems for screening are needed.

High-throughput cultivation is obviously only possible in conjunction with a miniaturization of the cultivation vessels. Usually this is achieved with the use of microtiter plate based technologies [8]. These technologies have been well established for heterotrophic cells and many commercial systems for integration into a liquid handling robot are available. They are widely adopted by industry and cultivation parameters and methods (aeration, growth, storage & replication) are well established [9,8]. Huber et al. demonstrated the capabilities of such a system by monitoring microbial growth and protein levels of cultures expressing a fluorescence protein after inductions of variable strengths using a commercial system (BioLector) and a Liquid Handling Robot [10,11]. Similar systems have not been reported for photothrophic organisms however.

We have developed a prototype for such a system for the automated cultivation of unicellular cyanobacteria by integrating a cultivation chamber with a particular design and construction into a liquid handling robot. The chamber can be fully handled by the robots manipulator arm. Cultures are kept in suspension within deepwell MTPs (DWPs) with controlled shaking, CO_2_ atmosphere, and light conditions. Each well can be treated as an individual photobioreactor, allowing for the parallel cultivation of currently up to 192 strains. The first prototype was included into a Tecan Genesis RSP 150 pipetting robot, and has been used to successfully screen for temperature tolerant clones out of a pool of enhanced strains [12]. Here we present an improved and expanded version of this previously developed prototype system. The screening platform is now integrated into a modern Tecan Freedom EVO 200 pipetting robot, with many analytic methods and systems available, and can be modified or extended in a flexible and modular manner.

For this study we used *Synechocystis* sp. PCC 6803 as the target organism, as it is a widely used model organism for phototrophic organisms and cyanobacteria in particular. Being from the morphological section *Chroococcales* it is unicellular and is officially classified as a fresh water strain, though it is highly tolerant to salt and marine media [13]. The *Synechocystis* sp. PCC 6803 genome was sequenced in 1996 [14], as the first genome from a photosynthetic organism.

## Results and discussion

This work describes a novel system for automated cultivation and screening of phototrophic microorganisms based on a pipetting robot with an in-house build and designed cultivation chamber.

The first section shows and discusses the various measured parameters and other methods which have been developed for the system, while the second section shows an example for an automated cultivation within the screening system.

### Established methods

#### Inoculation and cultivation conditions

Cultures can be inoculated into the screening system either by direct transfer from another liquid cultivation, such as a larger photobioreactor, or by picking single colonies from BG11 agar plates (either by manual picking or Genetix Genesis picking robot). An inoculation of monoclonal strains with a FACS System should also be straight forward, as some models allow direct sorting into microtiter plates.

Picking single clones from agar plates (or FACS sorting) allows having different monoclonal strains in each well of the DWP used for cultivation, which typically will have a lag phase of about 1 week.

Once inoculated into the wells, cultures have to be kept in suspension. Initial experiments (data not shown) demonstrated that *Synechocystis* cultures tend to aggregate quite strongly in the DWPs with simple orbital shaking, whereas a pattern of N-S & W-E shaking partially remedied this. However, as programmable linear shaking is harder and far more expensive to scale up, glass beads were tested to allow cultivation with orbital shaking. A growth test showed that the beads effectively keep the cultures in suspension, even with orbital shaking, whereas the number of beads (2-6) and their size (tested from 0.5 to 1.5 mm) seems to have little effect (data not shown). All cultivations presented within this work were performed with orbital shaking at 750 rpm, with two glass beads of 1.0 mm within each well.

The DWPs placed within the cultivation chamber have all important parameters (such as shaking, CO_2_, light and temperature) controlled and monitored. The temperature regulation and control is achieved by a heating mat below the DWP (see methods for details). In a setup with separate temperature sensors in each well and a set point of 28°C, the maximum deviation in any given well from the set point was ≤0.2°C while the maximum difference between any two wells was ≤0.3°C, demonstrating a very even and precise temperature control.

The cultivation system is also able to heat the cultures using complex predetermined temperature profiles. This allows the simulation of the cultivation conditions for other systems (e.g. large scale outdoor day-night conditions, including slow temperature ramp up during the day, and slow drop off during the night). Though for very exact temperature regulation using profiles a temperature probe inserted into a reference well is recommended. Complex temperature profiles have previously been successfully used in the prototype system for comparison of the thermal tolerance of various strains out of a pool of mutants [12].

Overall the cultivation can be performed in a semi-sterile fashion. The chamber and robot surfaces can be chemically sterilized, and the whole robot is covered by a hood. Only few contaminants are observed (microscopically) even during prolonged cultivation. If fully axenic cultures are required, specially constructed safety cells (which enclose the entire robot in a sterile environment) could be installed.

#### Cell density determination and absorption spectrum

**Figure 1:**
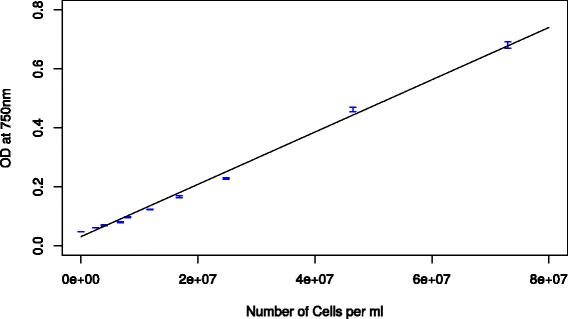
**OD**_**750**_ **as measured in 80 μl in 384-well-microtiter plates plotted over the number of cells per ml with manual counting.**

The optical density at 750 nm (OD_750_) of the cell cultures is an easily measured parameter which correlates with the amount of cells in suspension for unicellular organisms such as *Synechocystis*. Figure 1 shows the OD_750_ plotted over the cell count for a serial dilution of a *Synechocystis* culture. The obtained function for the linear regression was used for the calculation of cell counts (see methods). The resulting error coefficient (R = 0.996) demonstrates a good correlation of the parameters.

The lower detection limit is around 10^7^ cells per ml. For lower cell-densities the OD is not differentiable from a blank media measurement.

The measurement of full absorption spectra for cultures allows a quick and simple determination of pigment content and provides an impression of overall culture health, as phycobilly proteins are degraded during various stress conditions [15]. For some strains it might also be of interest to monitor this parameter to identify a shift in pigmentation, either due to environmental factors or because of genetic alterations. This could be of particular interest for screening optimal strains (or conditions) – e.g. for carotenoid production or similar.

**Figure 2:**
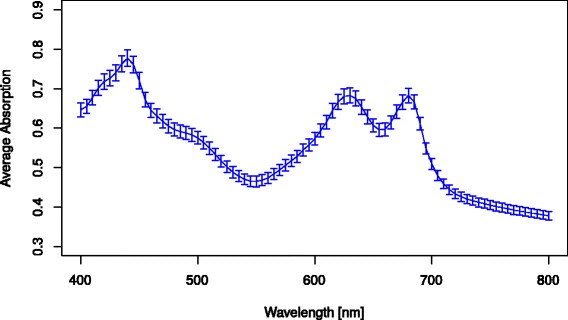
**Average absorption spectrum in the range of 400-800 nm for 86** ***Synechocystis*** **wt cultures normalized to OD**_**750**_ **= 0.4 after one week cultivation in the screening system.** Y-error bars indicate the standard deviation at each wavelength. 10 wells of 96 remained uninoculated and served as negative controls for cross-contamination.

Figure 2 shows the average absorption for *Synechocystis* cultures growing in different wells of a DWP after one week of cultivation in the automated system. The average relative standard deviation (RSD) over all cultures and wavelengths was 2.79%.

This deviation however is caused mostly by differences in the cell density of the various cultures; after normalization (see methods) the average RSD drops down to just 0.5%. This demonstrates a very similar pigmentation and fitness of all cultures in the screening system, irregardless of the position in the plate.

#### Chlorophyll a content determination

The extraction and measurement of chlorophyll a, which is a frequently used parameter for various purposes in cyanobacteria and microalgae biotechnology, can also be performed in a fully automated manner within the screening platform. The chlorophyll content can be used to approximate the cell-density and growth for some filamentous algae where an OD measurement is not possible, as long as they do not aggregate strongly during cultivation in the automated system and representative samples can be extracted by pipetting. However it has to be considered, that the total chlorophyll concentration is not only dependent on the number of cells but on their individual level of pigmentation, which is in turn affected by other factors such as culture vitality as well as nutrient and light conditions [16]. When used in addition to OD determination, the pigmentation of cells can be estimated.

**Figure 3:**
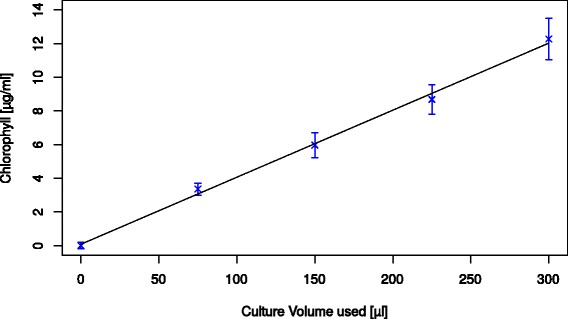
**Chlorophyll content for various dilutions of** ***Synechocystis*** **culture.** Y-error bars indicate the standard deviation between the 16 technical replicates (see methods) measured for each concentration.

Figure 3 shows the average chlorophyll a content for a serial dilution of *Synechocystis* culture. The average RSD between the technical replicates was at 9.2%. A linear regression of the dilution series however shows a very good correlation to the volume of culture used for extraction (R = 0.99).

To determine the standard deviation of biological replicates, chlorophyll a content was measured for 96 cultures after 11 days of cultivation in the screening system. The obtained RSD was 15.1%, showing an increased variation over technical replicates.

Overall chlorophyll content determination is not as precise as other parameters that can be measured and takes more processing steps and more time. Furthermore, for full automation a robotic centrifuge is required, which constitutes a significant additional investment. The possibility to measure this parameter when needed, however, shows the flexibility of the platform. If a lower variation is needed, more elaborated extraction or measurement protocols are probably recommended.

### Vitality determination

Another alternative to measure culture health is a cell based viability measurement adapted for use in the plate reader. The original method determines the viability of individual cells by measuring their chlorophyll and an unspecific green fluorescence in a microscope [17]. This method was adapted to obtain an average viability value for all cells within a given well, instead of a cell by cell viability determination.

**Figure 4:**
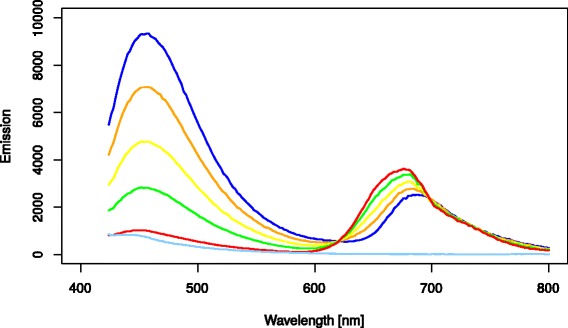
**Emission spectra from 420-800 nm for various mixtures of fully viable and non-viable** ***Synechocystis*** **cultures after excitation at 390 nm.** Mixtures range from 0% viable cells (dark blue) to 100% (red) in 25% increments. The blank (media) control is shown in light blue.

To identify the optimal wavelengths for use of this method in the plate reader, fluorescence emission scans were performed with various excitations on mixtures with differing percentages of viable and non-viable cells. The best results were obtained with an excitation of 390 nm (Figure 4).

**Figure 5:**
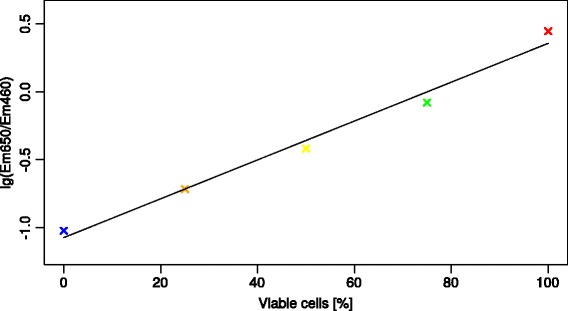
**Logarithm basis 10 of the Em**_**650**_ **/Em**_**460**_ **quotient values plotted over the percentage of viable cells and a linear regression of the data points.**

The emission peak at 460 nm clearly increases, while the chlorophyll emission at 650 nm drops, with the amount of non-viable cells. Figure 5 shows the logarithm basis 10 of the quotient of Em_650_ / Em_460_ plotted over the percentages of viable cells.

The regression error of R = 0.984 for the trend line demonstrates a good correlation for these parameters. This allows assigning cultures numerical “vitality value” from measuring just the emission at these two wavelengths that is independent of overall fluorescence intensity. Using the slope of the regression an equation can be calculated which will give a value between 0 and 100%, whereas 100% indicates a completely fit culture:

$$ Vitality\left[\%\right]=\frac{\left(\frac{E{m}_{(460)}}{E{m}_{(650)}}\right)+1.074}{0.0143} $$

Scans of cultures after a prolonged exposure to stress also resulted in a drop of this vitality value, because of the differing emissions of the stressed population, even if no cells were non-viable yet (data not shown). Therefore it is not possible to differentiate between these scenarios (general population of cells stressed vs some fit and viable and some not) with this method. However it allows for an easy and quickly measured parameter which should give a value of overall culture health and therefore of the potential productivity of the culture. If exact per cell determination is needed measuring samples under the microscope is required.

### MALDI-TOF-MS measurements

A MALDI-TOF-MS measurement can be used to detect secondary metabolites of interest when screening a range of strains. Also protein mass-fingerprinting for the identification of strains is possible or even culture health or similar parameters can be determined as long as markers and reference spectra are available. Hereby only a very small amount of cells (i.e. 80 μl of culture suspension from the DWP) and a very simple sample preparation is needed for the MALDI-TOF-MS measurement.

Direct loading of the MALDI-target from the robot screening platform allows integration into a complex high throughput MS screening pipeline applicable for screening of either a variety of different strains or of different cultivation conditions.

**Figure 6:**
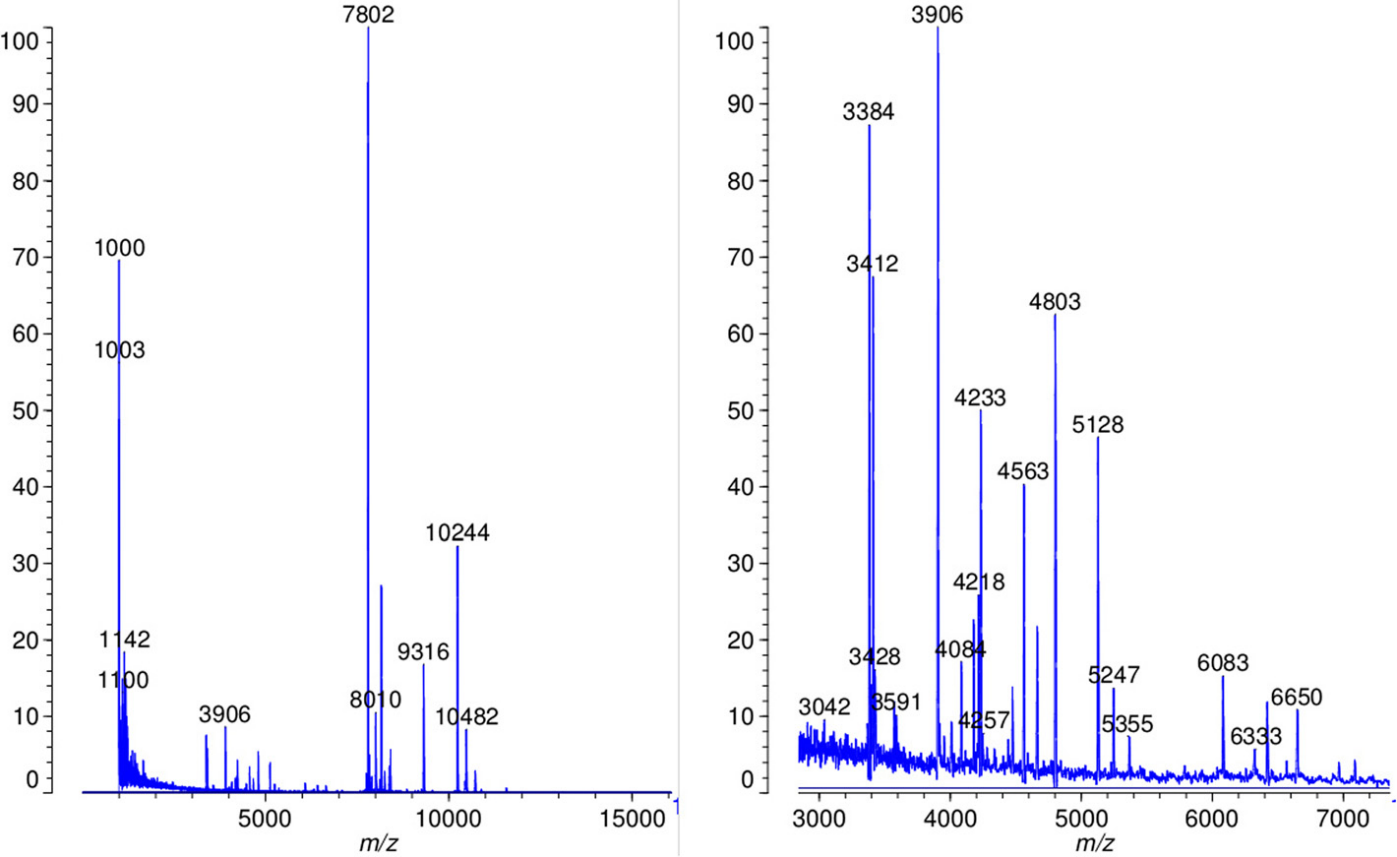
**MALDI TOF intact protein profiles of a** ***Synechocystis*** **wt culture cultivated in the microcultivation system.** Left side: full range up to 16000 m/z; Right: zoom in to region of interest in the range from 3000 - 7000 m/z. Measurements were obtained on a Shimazu Axima (confidence in linear mode; integration of 24 individual profiles).

The best results for intact protein profiling were obtained after incubating the sample for at least 30 minutes in a matrix of sinapinic acid. Figure 6 shows an example spectrum obtained from a culture growing in the microcultivation system for 9 days (well A7 from the cultivation experiment discussed later).

For this proof of concept we could show that direct loading of MALDI targets is useful to produce a complex spectrum of protein signals for further analysis. Depending on the strain(s) used and the applications the protocol might have to be adapted or changed, however.

### Culture preservation

Two methods have been established for the automated backup of cultures in the HTS system.

For short or mid-term backup, cultures can be transferred into 96 well plates prepared with BG11 agar. Depending on humidity, temperature, and light conditions they can be stored for up to several weeks (just as regular plated cultures). Cultures can then be resuspended by the liquid handling robot and used to directly reinoculate DWPs or transferred to a new agar plate for continued preservation.

All re-inoculated wells demonstrated exponential growth after 2 days (data not shown). This method allows a quick retrieval of cultures of interest, either for upscaling or for repetition of experiments.

For long term storage the liquid handling robot is able to prepare cryopreservation samples of cultures. Storage has been tested for up to 6 months (with a success rate of 100%), but can presumably be extended almost indefinitely, the retrieval process however is more labor intensive (see methods).

### Automated cultivation

Our cultivation chamber, with the established parameters, allows an automated cultivation of phototrophic microorganisms.

In all experiments we had control wells, which were not inoculated but instead contained only medium. They did not show any increase in OD, which demonstrates that cross contamination between wells does not take place, therefore each well can be treated as an individual photobioreactor.

A fed batch cultivation with periodic dilutions was performed. The average growth rate calculated for each well in the periods between dilutions were comparable to those obtained in cultivation in larger photobioreactors (data not shown). However at high cell densities they are not constant and drop off.

One possible explanation for this behavior is due to energy or nutrient limitation, such as light or CO_2_ (even though light intensity was scaled with cell growth - see methods). This could be limited if the cultivation parameters are adapted to their optimal values for each given OD during the course of the experiment. The optimal settings for some parameters such as mixing and CO_2_ concentration however can be challenging to predict, are typically empirically determined, and vary depending on various additional factors (e.g. the strain being cultivated).

An alternative cultivation method which avoids many of these problems is a semi-continuous cultivation. Here the OD is kept almost constant, by automatically diluting the culture back to a set value at least once a day. Growth rates can again be calculated by the OD increase in between each dilution but also over longer periods of time under consideration of the average dilution rate. The main advantage of this cultivation type is that limiting conditions arising over the course of a fed batch cultivation (e.g. mixing, light, or CO_2_) can be excluded and the effect of various conditions can be more precisely and reproducibly examined. It also allows for longer cultivations in order to investigate potential long term effects of for selection-for-growth-approaches. Therefore we recommend this form of cultivation if such applications are intended. However, it comes at the cost of more consumables and somewhat increased processing time, which might become a bottleneck for setups with greatly increased throughput (i.e. thousands of parallel cultures, instead of 192).

**Figure 7:**
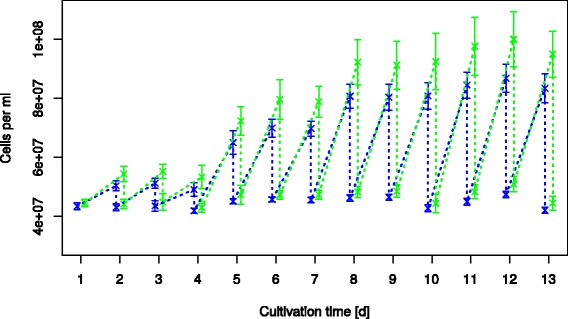
**Cultivation over 13 days with daily dilutions back to the starting OD of 0.4.** The cell count at the various wells (edge of the plate (green) & middle wells (blue)) are shown over the cultivation time. Light intensity was varied during the cultivation time [photon flux in μmol m^-2^ s^-1^]: Day 1-3: 15; Day 4-7: 32; Day 8-10: 51; Day 11-13: 72.

**Figure 8:**
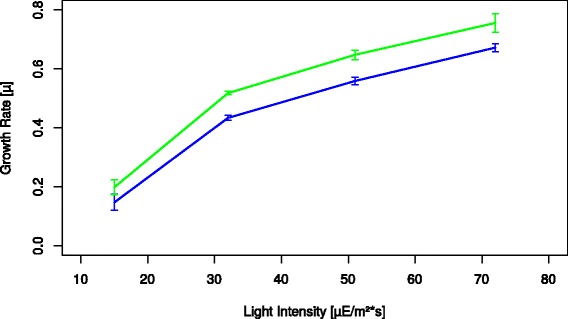
**Average growth rate of cultures (edge wells (green); middle wells (blue)) at the four tested light intensities.** Error bars at each value represent the difference in the average growth rate of the three days tested.

Figure 7 shows the results for a cultivation experiment over 13 days with daily culture dilutions and four distinct light intensities, while keeping the other parameters constant. The results show how a daily automated dilution can be used to keep the cultures at reproducible conditions. The edge-effects (cultures in the middle wells of the plate showing different growth than those at the edge wells) clearly visible in Figure 7 (and Figure 8) are discussed further bellow.

The average vitality value throughout the whole cultivation was 96.9% with an average RSD of 2.16%, demonstrating good cultivation conditions with low variance of culture vitality between wells.

Varying the light intensity was chosen as an example, to demonstrate the effect of cultivation conditions on the growth characteristics of the strain(s) being cultivated, and to show how the presented system allows to easily generate high precision, reproducible results. At each light intensity the cultures demonstrated very similar growth rates during the three days of cultivation (see Figure 8).

The results show a very high reproducibility across the three days tested at each intensity. As expected the growth rate increases with increasing light. Even at a photon flux of 72 μmol m^-2^ s^-1^ (maximum current output of the cultivation chamber) the growth of cultures does not appear to be fully light saturated, at least not at the higher ODs reached before dilution. This indicates that for maximal growth rates or light inhibition experiments either a stronger light source, a lower starting cell density, or more frequent dilutions are needed.

**Figure 9:**
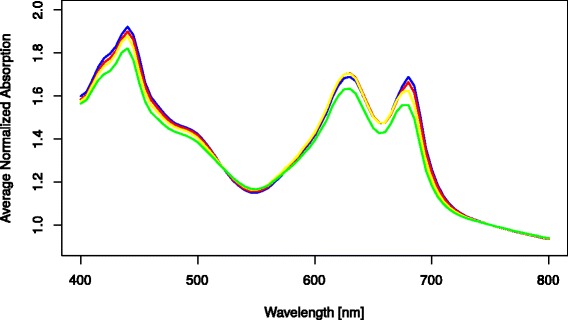
**Average normalized absorption spectra for the various stages of cultivation.** The values shown are always at the last day at a given light intensity [photon flux in μmol m^-2^ s^-1^]: 15 (blue); 32 (red); 51 (yellow); 72 (green).

The normalized absorption spectra for the first three stages of cultivation (see Figure 9) are very constant. Meanwhile at a photon flux of 72 μmol m^-2^ s^-1^ a noticeable drop in pigmentation of the cultures is visible. This is to be expected as the cells reduce their pigmentation at higher light intensities to avoid light inhibition. The relative standard deviation between the various wells was only 0.6% for 15 μmol m^-2^ s^-1^ photons and 0.5% for the other three intensities, demonstrating very homogeneous pigmentation across the various cultures in the wells of the DWP.

When using MTPs for cultivation it has to be considered that there appears to be a significant difference between wells at the edge of the plate with access additional light (due to light entering from the side of the semi-transparent DWP) to those in the middle of the plate which are shaded by the surrounding wells (Figure 7). Those in the middle thus have a slightly lower growth rate (Figure 8).

Welch’s t-test was used to calculate the statistically significance of these differences. The average t-value for all determined growth rates when comparing these two groups was 7.02 with two degrees of freedom. This translates to a p-value of 0.0197 indicating a statistically significant difference between cell growth rates for these two groups of wells. It might be of interest to note however, that the variance in OD of the various wells (error bars in Figure 7) is greater than the variance in the growth rates. This is due to the fact that for the automated dilutions OD is calculated as if it were a completely linear correlation, which is only an approximation, due to OD being based on light scattering, not absorption. Dilutions down from higher OD wells will typically have a higher OD after dilution than those from lower OD wells. Therefore ODs in wells with a slightly increased growth rate will drift upwards over time.

Meanwhile the vitality measurement and the normalized absorption spectra showed no statistical difference between the wells at the edge of the plate and the rest (p- value of 0.4952 and 0.6858 respectively).

In general the difference in growth between edge wells and the rest means, that depending on whether it is relevant for the analysis being performed (i.e. looking for small differences in growth), the edge wells should be either excluded or treated as a separate group. Meanwhile the relative standard deviation between wells not on the edge of the plate is typically only around 5% (see error bars for middle wells in Figure 7). If various replicates are made it is important to spread them to different random positions for more robust results. For other analysis (e.g. compound screening) the slight differences in growth rate are probably not relevant.

As mentioned above the most likely cause for the observed difference between wells and the edge and the middle of the plate is a slightly higher light availability. Therefore further innovation in the design of the chamber, or the DWPs, could reduce (or eliminate) this effect. The difference in growth might also be reduced by cultivating at (or near) light saturation (but before significant inhibition), as slight differences in light intensity are then expected to have a less noticeable effect on culture growth.

## Conclusions

The results presented here demonstrate the possibility of high throughput culturing and screening of a unicellular cyanobacterium in a flexible automated platform. The platform should be applicable for the cultivation of many other phototrophic microorganisms as is, and be adaptable for even more. Various methods (OD, absorption spectra, chlorophyll content, vitality, MALDI-MS, agar- , and cryo-conservation) have already been established to analyze the cultures within the screening platform. However these represent only a small subset of the possibilities, as the flexible nature of the system allows for the easy integration of many additional methods, especially if an assay in a MTP-format is available (e.g. biochemical or enzymatic assays, qPCR, ddPCR, Luminex, HPLC…).

The prototype in-house cultivation chamber produced reproducible and consistent results with low variability between most wells. As there is no cross contamination between wells, each can be treated as an individual miniaturized photobioreactor. The semi-continuous cultivation presented allows experiments for extended periods of time with constant and reproducible results. While the cultivation in the current system is limited to 192 cultures at a time, it should be straightforward and relatively cost efficient to scale further up, allowing the cultivation of thousands of cultures in parallel.

Since such a HTS system has previously not been available for phototrophic microorganisms it opens the possibility to screen a relatively untapped pool of organisms and metabolites, with applications in many fields (e.g. pharmaceuticals, biofuels, ecology).

## Methods

### Organisms

- *Synechocystis* sp. PCC 6803 wild type, provided by Cyano Biotech Berlin GmbH, Germany

### Medium

All strains were cultivated in sterile marine BG11 medium (modified from [18]): 30 g/l instant ocean (Aquarium Systems, rue Gambette 43, F-57400 Sarrebourg, France); 17.65 mM NaNO_3_, 0.18 mM K_2_HPO_4_, 0.03 mM citric acid, 0.003 mM EDTA, 0.19 mM Na_2_CO_3_, 0.03 mM ferric ammonium citrate and trace metals (2.86 mg/l H_3_BO_3_; 1.81 mg/l MnCl_2_.4H_2_O; 0.22 mg/l ZnSO_4_.7H_2_O; 0.390 mg/l Na_2_MoO_4_.2H_2_O; 0.08 mg/l CuSO_4_.5H_2_O; 0.05 mg/l Co(NO_3_)_2_.6H_2_O ).

### Monoclonal picking

Cultures were prepared by plating 500 cells on BG11-1%-Agar plates. After about 2 weeks at room temperature single colonies were either automatically picked with a Genetics Qpix (manual inverted threshhold mask with center frame of 60 to 95) or picked by hand with a sterile loop and transferred into 1.5 ml mBG11 in 96-Deepwell plates (DWPs).

### Liquid handling robot

Automated screening was performed on a Tecan Freedom Evo 200 pipetting Robot. It is equipped with a RoMa (Robotic Manipulator Arm) and a LiHa (Liquid Handling Arm) with 1 ml dilutors. The worktable is equipped with carriers for disposable tips, MTPs and 1.5 ml reaction tubes. Tempered carriers (with a Haake DC30-K20; -28°C to 100°C) are available for MTPs, 1.5 ml reaction tubes, and 100 ml throughs. Additionally the robot is equipped with a Tecan Infinite M200 Pro plate reader, a Te-Shake tempered shaker, a Hettich Rotana 460 RSC centrifuge and a custom build cultivation chamber for cultivation of phototrophic microorganisms.

### Design of the automated cultivation platform

**Figure 10:**
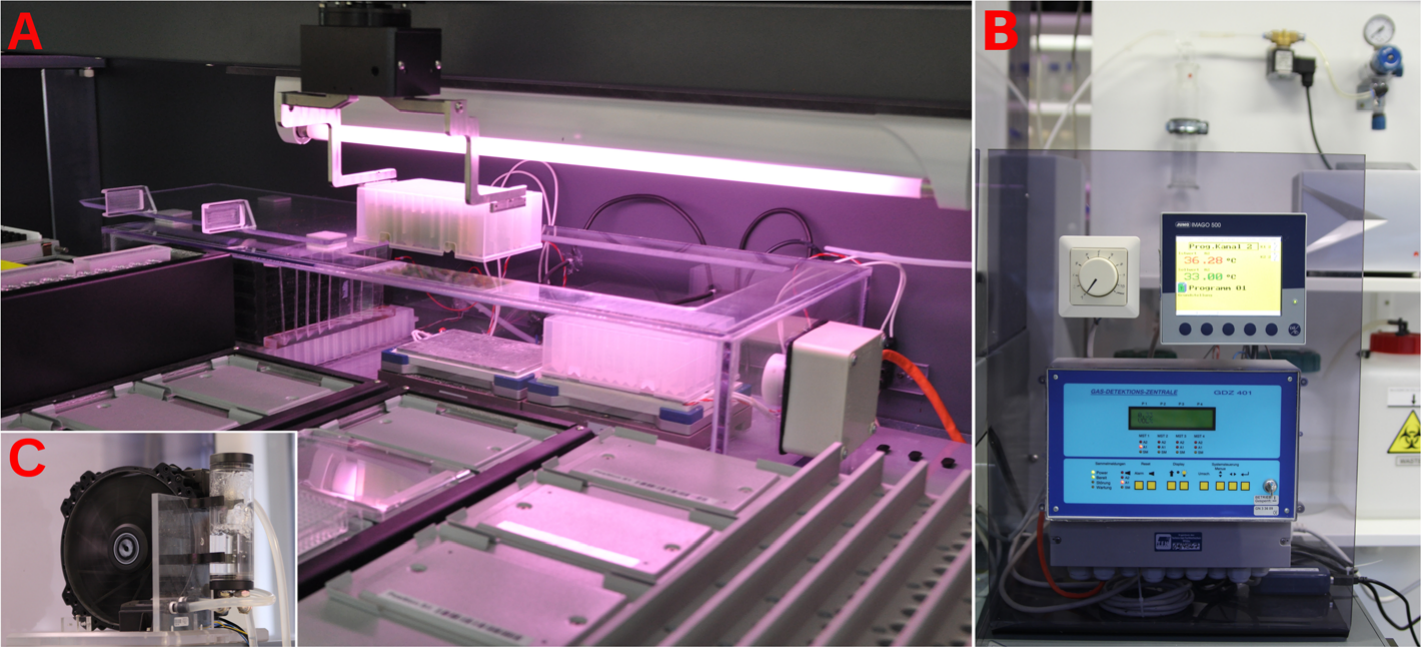
**The system for automated cultivation consists of: The cultivation chamber integrated in the robot (A); an external control panel (B); and an external radiator (C).**

The cultivation chamber (Figure 10) has been especially designed for the cultivation of phototrophic microorganisms inside the Tecan Freedom Evo liquid handling Robot. It allows fully automated cultivation with low risk of contamination. It consists of a custom build polycarbonate chamber, dimmable lamps (Aquamedic FHO 24 W Plant Grow), a gas control unit (SCENTY GDZ 401), a CO_2_ sensor (GSI 400B), a pressure regulator, a solenoid valve, a gas diffuser, two programmable shakers (Variomag Teleshake), a heating system (Jumo Imago 500 with custom heating foils by Friedr. Freek GmbH), and two radiators (internal: Magicool double power 80 mm with two 80 mm fans; external: Magicool 180 mm with two 200 mm fans) connected with a pump (Laing DDC-1 T).

The main compartment has an inlay-lid with special holders for the RoMa grippers which allow the robot to place the lid to the side (Figure 10A), before retrieving the DWPs from the shakers. After placing the DWPs on a carrier outside of the cultivation box, they are reachable by the LiHa arm, allowing for any analysis available to the platform. The sloped edges of the inlay-lid allow easy and precise placement by the robot. A CAD File of the chamber housing is provided as Additional file 1.

Illumination is placed above the chamber. Two fluorescent tubes are arranged lengthwise above to cultures to allow for even light distribution. The light intensity can be varied (photon flux of 1.5 – 73 μmol m^-2^ s^-1^) using the dial in the external control panel (Figure 10B).

The shakers in the chamber are fully programmable and allow linear and orbital shaking (or even combinations thereof) from 100-2000 rpm. They have been retrofitted with a heating foil which is isolated from the shakers themselves with cast gypsum. An aluminum plate on top of the foil allows even distribution of the heat to the DWP wells. The connected temperature control unit is integrated in the external panel (Figure 10B) and allows PID-controlled set temperatures or full temperature profiles. The temperature can be measured with a sensor integrated in the heating foil layer (which is sufficient for set point temperature) or with a PT100 sensor placed in a reference well for more exact measurements. To allow temperature regulation down to lower temperatures and to avoid a heat buildup within the chamber, a radiator and fans have been inserted. The vents in front of the fans stop direct air circulation onto the DWPs to avoid uneven tempering. This internal radiator is connected to a larger external one (Figure 10C) which is used to dissipate the heat.

The CO_2_ sensor is fitted to the side of the chamber via a threaded opening. The gas control unit is placed on the external control panel (Figure 10B). If the set CO_2_ value falls below the set point (0-5%) the control unit opens the solenoid valve which lets 10% CO_2_ air mixture at 0.2 bar through to the gas diffuser. The gas diffuser bubbles the air through a column of water to increase its humidity, before passing a 0.22 μm sterile filter (PVDF, Carl Roth) and reaching the chamber.

All wiring is connected to luster connector strips which allows an easy removal of the chamber from the robot for sterilization.

### Evaporation refill

The volume in each well of the DWPs was measured using disposable Tecan conductive tips. The difference to the set culture volume (typically 1.8 ml) was then calculated and replaced with sterile dd-water. This was made before any other measurement took place to avoid distorting results due to evaporation.

### Optical density

Optical density measured at 750 nm is a parameter often used to determine cell density for unicellular cyanobacteria or microalgae. OD_750_ was determined by transferring 80 μl of sample into a transparent 384 well MTP (leaving one row and one column empty between each sample) and measuring the OD_750_ (5 flashes) in a Tecan Infinite M200 Pro plate reader.

### Cell quantification

To calculate the cell density in cells per ml from the OD_750_ a calibration is needed. A log-phase *Synechocystis* culture, was serially diluted in 3:2 steps to create a total of 11 concentrations. The cell count for each dilution was manually quantified under a microscope using an improved Neubauer counting chamber and measured in the plate reader with 8 replicates (one MTP column).

The following formula was thus determined and used for all calculations from the measured OD_750_ value to the cell density:

$$ Vitality\left[\%\right]=\frac{\left(\frac{E{m}_{(460)}}{E{m}_{(650)}}+1.074\right)}{0.0143}. $$

### OD normalization

To normalize cultures to a specific OD, the current OD was first measured to calculate the volume of culture needed to reach the wanted OD:

$$ Volum{e}_{Culture}=\frac{{\mathrm{OD}}_{\mathrm{target}}}{{\mathrm{OD}}_{measured}}*{\mathrm{Volume}}_{\mathrm{target}}. $$

The calculated volume was then transferred to a new sterile DWP preloaded with glass beads (see cultivation conditions below). The new DWP with normalized OD was used for all other measurements (post-dilution OD, vitality, absorption spectrum, chlorophyll a content, MALDI).

### Cultivation conditions

Cultures were kept in a 96-deepwell plate inside the custom cultivation chamber. Some wells contained only sterile media, and were used as a control for the detection of potential cross contaminations. Cultures were kept in suspension by orbital shaking at 750 rpm. To avoid cell aggregates and cell adhesion to walls of the DWP, three glass beads (1 mm ø) were added to each well. The atmosphere was set to 2% CO_2_. Cultures were incubated at constant 28°C. Volume loss due to evaporation was refilled every day with sterile dd-water (200-350 μl).

For the periodically diluted cultivation the light intensity was increased depending on cell density (3 μmol m^-2^ s^-1^ photons first 2 days after inoculation; up to 72 μmol m^-2^ s^-1^ photons at the end). With regular dilutions back to OD = 0.2.

For the semi-continuous cultivation the cultures were kept in the cultivation chamber for 5 days to allow full acclimatization. Four different light intensities (15, 32, 50 & 72 μmol m^-2^ s^-1^ photon flux) were then tested for three days each, while the cells were diluted daily back to the set OD of 0.4. For dilution down from higher ODs the set point was set to 0.39 and 0.35 (days 5-9 & 19-13 respectively) to allow the dilutions to reach closer to the target OD.

### Growth rate calculation

The growth rate (μ) was calculated for each strain at any given temperature by comparing the OD_750_ at the time point 0 (X_0_) with OD_750_ after time t (X_t_):

$$ \upmu = \ln \frac{X_t}{X_0}\cdot \frac{1}{t} $$

### Absorption spectrum

The absorption spectrum was measured in a range from 400 to 800 nm using 5 nm steps and two flashes in the integrated Infinite M200 Pro reader. For normalized representations each value is divided by the absorption (/OD) value at 750 nm:

$$ \mathrm{Normalized}\ {\mathrm{Abs}}_x=\frac{{\mathrm{Abs}}_x}{{\mathrm{Abs}}_{750}} $$

### Chlorophyll a content determination

Chlorophyll a was extracted based on a protocol described by Marsac et al. [16]. 300 μl of sample were transferred into 96 well MTP, then centrifuged for 10 min at 3,500 g at 4°C. The supernatant was then removed, leaving 30 μl behind. The sample was then resuspended by pipetting up and down (10-times 25 μl). After adding 270 μl of methanol (100%) and a second mixing step (5-times 270 μl), the plate was covered with a dark lid, and incubated for one hour on a MTP carrier cooled to 4°C. The denatured sample was then centrifuged again for 10 min at 3,500 g at 4°C. 80 μl supernatant were then transferred to a 384 well MTP and the absorption at 650 nm was measured. The following formula gives the chlorophyll concentration in μg/ml:

$$ \mathrm{Chlorophyll}\ \mathrm{a}\left[\upmu \mathrm{g}/\mathrm{ml}\right]=\frac{{\mathrm{Abs}}_{650\mathrm{nm}}*\ 14.3}{0.3 ml} $$

Whereas the factor of 14.3 was previously determined using samples of known concentration.

A serial dilution of culture samples with 16 replicates each (two MTP columns) was made to determine the technical reproducibility. For this 300, 225, 150 and 75 μl culture (OD = 1.2), as well as 300 μl medium (blank), were pipetted into a 96 well MTP and chlorophyll extracted and measured as described above.

### Vitality measurement

To quickly determine the vitality of *Synechocystis* cultures a new method based on a microscopic method for viability determination [17] was used.

To determine the optimal settings emission scans from 400-800 nm (culture OD = 0.2; 2 nm steps, 2 flashes; manual gain set to 100; 20 μs integration time) were performed with excitations of 380, 390, 400, 410, 420, 430 & 440 nm. A 100% viable cell culture (log growth culture) and a 0% viable cell culture (3 days incubation at 50°C) were mixed to obtain cultures with 0, 25, 50, 75 and 100% viable cells. The content of viable cells was confirmed with microscopic imaging as described by Schulze et al. [17].

Optimal parameters were found to be emission measurements at 460 nm and 650 nm after an excitation at 390 nm (two flashes; manual gain set to 100; 20 μs integration time). A numerical vitality value in percent can be calculated with the following formula, which was determined by mixing cultures of 0% and 100% health (see results):

$$ Vitality\left[\%\right]=\frac{\left(\frac{E{m}_{(460)}}{E{m}_{(650)}}\right)+1.074}{0.0143} $$

### MALDI-TOF-MS

To record a mass spectrum of culture extracts, 80 μl culture suspension at around OD = 0.4 (same plate used for post-dilution measurements) were centrifuged down at 3,200 g. Supernatant (75 μl) was then removed and the pellet resuspended in 40 μl matrix (25 mg/ml sinapinic acid; 50% acetonitril; 0.5% trifluoroacetic acid). After at least 30 min incubation 3 μl of the resulting extract were pipetted onto a MALDI-TOF-MS target plate pretreated with EtOH saturated with sinapinic acid and placed on a special carrier within the robot. For automated loading the sample onto the MALDI target with the robots LiHa arm (with 200 μl DITIs (tips) and 1 ml dilutors) a very low distance to the target, a special liquid-class with a low dispense speed (10 μl/s) and high conditioning and excess volume is needed. The loaded target was then manually placed in the Shimazu Axima MALDI-TOF-MS.

### Agar backups

96-well agar plates were created by transferring 100 μl of liquid 1% BG11 Agar (kept at 60°C in the tempered robot carrier) with pre-warmed tips into each plate well with the LiHa. The plate was then sealed, and allowed to cool at room temperature solidifying the agar.

To inoculate the plates, 10 μl of culture (OD = 0.4) were automatically transferred onto the plate, mirroring the well positions of the DWP. The plate was sealed with a gas permeable foil (Greiner Breathseal), and stored at low light (2 μmol m^-2^ s^-1^ photons) at room temperature.

For re-inoculation 150 μl medium were added by the robot and mixed on top of the agar. The plate was then transferred into the cultivation chamber and incubated overnight at 750 rpm and a photon flux of 15 μmol m^-2^ s^-1^, allowing the cells to fully go into suspension. After retrieval from the chamber the 150 μl inoculated medium were transferred into a DWP preloaded with 850 μl medium. The cultures were then cultured as previously described, increasing the volume back to 1.8 ml once logarithmic growth began (after 2 days).

### Cryo-conservation

To preserve cultures, a modified protocol based on the standard DMSO cryopreservation of cyanobacteria [19] was used. 150 μl were automatically transferred out of each well of the DWP plate, and transferred into a regular 96-well MTP placed on a carrier cooled to 4°C. After 30 min incubation 150 μl 14% DMSO were added and immediately mixed with the sample by pipetting up and down. The MTP was then sealed with a PCR foil and manually transferred to -80°C for quick freezing and storage.

For retrieval cultures are manually plated on BG11 agar plates.

### Statistical analysis

To determine if there were statistically significant differences between the measurement results of the various parameters for cultures in different wells, a two tailed test was used. First Welch’s t-test [20] was used to determine the t-value:

$$ t=\frac{\left(\overline{X_1}-\overline{X_2}\right)}{\sqrt{\frac{s_1^2}{n_1}+\frac{s_2^2}{n_2}}} $$

$$ \overline{X_i} $$… sample mean

*s*_*i*_… sample variance

*n*_*i*_… sample size

then the Welch–Satterthwaite [21] equation was used to estimate the degrees of freedom:

$$ DF\approx \frac{{\left(\frac{s_1^2}{n_1}+\frac{s_2^2}{n_2}\right)}^2}{\frac{s_1^4}{n_1^2*{\nu}_1}+\frac{s_2^4}{n_2^2{\nu}_2}} $$

where

$$ {\nu}_i={n}_i-1 $$

Using these parameters the p-value could be determined using a t-table.

## Additional file

Additional file 1:
**CAD Design for the cultivation chamber housing and the inlay-lid as in STL Format.** Longest edge of the chamber is 600 mm.
